# Developmental mechanisms underlying pediatric epilepsy

**DOI:** 10.3389/fneur.2025.1586947

**Published:** 2025-06-03

**Authors:** Vishal Lolam, Achira Roy

**Affiliations:** Neurodevelopment Laboratory, Neuroscience Unit, Jawaharlal Nehru Centre for Advanced Scientific Research, Bengaluru, India

**Keywords:** epilepsy – abnormalities, pediatric epilepsy, SUDEP, neurodevelopment, drug resistance, Cilia, sleep and circadian rhythm, PI3K-AKT–MTOR pathway

## Abstract

Pediatric epilepsy affects a large proportion of children, with a huge variability in seizure onset. Due to complicated etiology, wide range of associated comorbidities, and difficulty in obtaining clear physiological data from children, epilepsy management in pediatric patients often poses a critical challenge. Importantly, around 30% of these patients remain non-responsive to current anti-seizure drugs and develop a higher risk of developmental and cognitive delay and, in worse situations, premature death. One of the key treatment methods currently used for drug-resistant epilepsies is surgical resection of the epileptic foci. However, such patients often develop new epileptic foci post-surgery. This, in turn, enhances the need for recurrent invasive brain surgeries, impairing the overall quality of life in these children. Thus, mechanistic understanding of different types of pediatric epilepsy is critical to discovering more targeted molecular approach(es). For a long time, the occurrence of epilepsy was considered solely due to the abnormal functioning of single ion channels. However, in recent years, a huge number of genetic and non-genetic (environmental) factors have been associated with different types of pediatric epilepsy. Clinical diagnoses, coupled with a basic understanding of molecular and cellular mechanisms using different model systems, have been instrumental in unraveling new avenues for modern non-invasive targeted pharmacological therapies. Yet, the field has just started to evolve, and many challenges and contradictory hypotheses still exist. This comprehensive review discusses underlying developmental mechanisms associated with pediatric epilepsy. Specifically, we highlight how the PI3K-AKT–MTOR pathway acts as a critical node interconnecting the diverse mechanistic strategies, that may eventually help overcome the seizure burden in the future.

## 1 Introduction: epilepsy in children

Epilepsy is among the most common neurological disorders affecting people of all age groups (1, 2). As defined by the International League against Epilepsy (ILAE), epilepsy is one meeting any of the following conditions: (a) at least two unprovoked seizures occurring >24 h apart; (b) one unprovoked seizure and a probability of further seizures similar to the general recurrence risk after two unprovoked seizures; and (c) diagnosis of an epilepsy syndrome (3–5). Epileptic seizures are very distinct from just any other non-epileptic seizure event in the sense that epileptic seizures occur due to abnormal, excessive, or simultaneous activity of “neuronal populations” in the brain, which may or may not have any clinical manifestations. Epilepsy syndromes manifested in children are especially complicated, overlapping in nature, and multi-faceted to understand. Children with early-onset epilepsy are highly predisposed to developmental and cognitive delay and sensory-motor abnormalities since the critical period for many neurological functions lies in childhood (6, 7). Further, about 30% of patients suffering from childhood epilepsies are intractable to any regimen of current medications (8–10). The combination of associated comorbidities and drug resistance negatively influences the patient’s quality of life and is also potent in elevating their risk of premature death, creating a huge emotional burden on caregivers (11–13). Therefore, to fight this challenge and identify potential non-invasive therapeutic avenues, studying the underlying mechanisms driving pediatric epilepsy is critical.

Historically, epilepsy has always been considered a channel protein manifestation. However, advancements in genetic screens have identified diverse epilepsy-causing variants, ranging from mutations in cellular signaling pathways to the components of circadian rhythm (14–19). In this review, we will discuss the model systems that helped in identifying the molecular mechanisms underlying pediatric genetic epilepsies beyond channelopathies and how these models can be instrumental in discovering potential treatment strategies. Further, we strongly emphasize the ongoing challenges and controversies in the field. Certain common terminologies used throughout the text are defined in Table 1.

**Table 1:** Common terminologies in the field of epilepsy [Adapted from Fisher et al. (3)].

Term	Definition
Seizure	Transient symptoms due to abnormal excessive or simultaneous activity of a neuronal population in the brain, with or without any clinical manifestations
Epilepsy	A disease of the brain defined by any of the following conditions: (1) At least two unprovoked (or reflex) seizures occurring >24 h apart; (2) one unprovoked (or reflex) seizure and a probability of further seizures similar to the general recurrence risk (at least 60%) after two unprovoked seizures, occurring over the next 10 years; (3) diagnosis of an epilepsy syndrome. Epilepsy is resolved for individuals who have an age-dependent epilepsy syndrome but are now past the applicable age or those who have remained seizure-free for the last 10 years, with no antiseizure medicines for the last 5 years
Epileptogenesis	A process that includes mechanisms driving functional, structural, or network reorganization changes in the brain that may lead to the development of, or progression of, spontaneous seizures and epilepsy.
Status epilepticus	Status epilepticus occurs when a seizure lasts more than 5 min or when seizures occur very close together, and the person doesn’t recover consciousness between them.
Focal seizures	Originating within networks limited to one hemisphere. They may be discretely localized or more widely distributed.
Generalized seizures	Originating at some point within, and rapidly engaging, bilaterally distributed networks
Autonomic seizures	A distinct alteration of autonomic nervous system function involving cardiovascular, pupillary, gastrointestinal, sudomotor, vasomotor, and thermoregulatory functions
Tonic	A sustained increase in muscle contraction lasting a few seconds to minutes
Atonic	Sudden loss or diminution of muscle tone without apparent preceding myoclonic or tonic event lasting ~1–2 s, involving head, trunk, jaw, or limb musculature.
Clonic	Jerking, either symmetric or asymmetric, that is regularly repetitive and involves the same muscle groups
Myoclonic	Sudden, brief (<100 msec) involuntary single or multiple contraction(s) of muscles(s) or muscle groups of variable topography (axial, proximal limb, distal). Myoclonus is less repetitive and less sustained than clonus
Tonic–clonic	A sequence consisting of a tonic followed by a clonic phase
Generalized tonic–clonic (GTC)	Bilateral symmetric or sometimes asymmetric tonic contraction and then bilateral clonic contraction of somatic muscles, that are usually associated with autonomic phenomena and loss of awareness. These seizures engage networks in both hemispheres at the start of the seizure
Atypical absence seizures	An absence seizure with changes in tone that are more pronounced than in typical absence or the onset and/or cessation is not abrupt, often associated with slow, irregular, generalized spike–wave activity
Typical absence seizures	A sudden onset, interruption of ongoing activities, a blank stare, possibly a brief upward deviation of the eyes. Usually, the patient will be unresponsive when spoken to. Duration is a few seconds to half a minute, with very rapid recovery
Febrile seizures (FS)	Seizures occurring in pediatric patients between 6 and 60 months of age, triggered by fevers higher than 38°C (≥100.4°F), without any known underlying medical condition such as trauma, CNS infection, neurodevelopmental disorders, genetic mutation, afebrile seizures or predisposition to epilepsy.

## 2 Article types

This is a review article that comprehensively discusses different mechanistic aspects of pediatric epilepsy, using different model systems and finishing with newer avenues of prospective therapies and existing gaps. No specific databases have been used in this manuscript.

## 3 Current classification of pediatric epilepsy

Epilepsy is a multifactorial disorder, caused by genetic and/or environmental factors and is classified into different types, each with its own etiology, physiological properties, and onset (20). Differential diagnoses aid clinicians in classifying the type of epilepsy the patient is experiencing, which in turn may help ascertain the therapeutic strategy. Historically, epilepsy classification heavily relied on the clinical symptoms of the patients. However, this classification system has recently undergone a significant change from a symptom-based approach to a more sophisticated, multidimensional framework. A critical feature of this change is the integration of neuroimaging findings, genetic screen data, and etiology (21). The current classification system is a hierarchical three-level scheme (21). It begins with classifying a seizure type into focal, generalized, and of unknown onset/unclear origin, followed by classifying the epilepsy type, eventually categorizing as epilepsy syndromes. An epilepsy syndrome is defined as, “a characteristic cluster of clinical and electroencephalography (EEG) features, often supported by specific etiological findings” (4). It has a distinct set of comorbidities, etiology, and age of onset, and often has direct consequences for treatment and prognosis.

**Figure 1:**
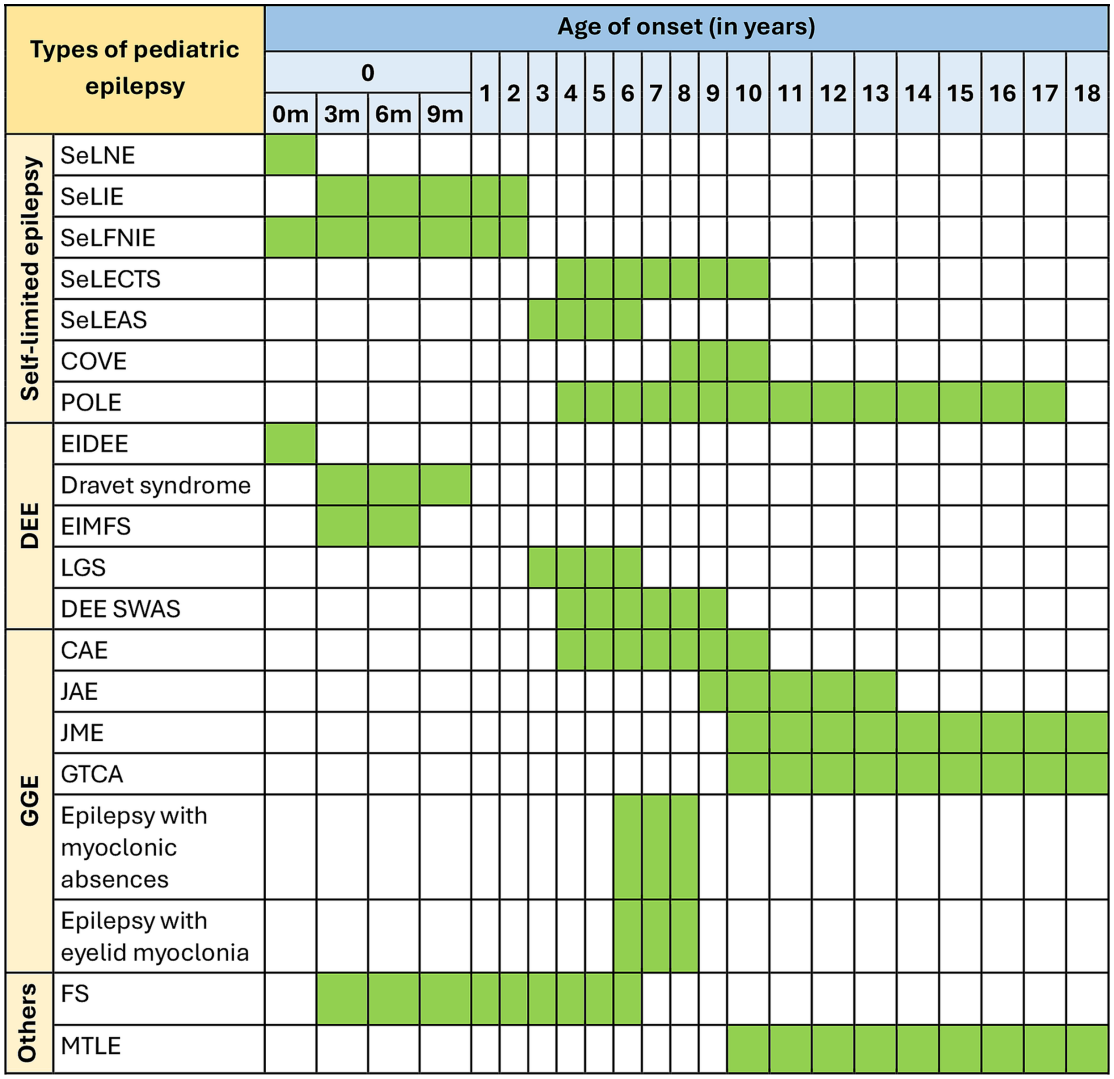
Seizure onset for different pediatric epilepsies. Green blocks represent the age range in which different types of pediatric epilepsy show seizure onset. The first year of age (0–1 year) has been subdivided into 0 m, 3 m, 6 m, and 9 m of age (m, months). SeLNE, self-limited neonatal epilepsy; SeLIE, self-limited infantile epilepsy; SeLFNIE, self-limited familial neonatal-infantile epilepsy; SeLECTS, self-limited epilepsy with a centrotemporal spike; SeLEAS, self-limited epilepsy with autonomic seizures; COVE, childhood occipital visual epilepsy; POLE, photosensitive occipital lobe epilepsy; EIDDE, early infantile developmental and epileptic encephalopathy; EIMFS, epilepsy of infancy with migrating focal seizures; LGS, Lennox–Gastaut syndrome; DEE SWAS, developmental and/or epileptic encephalopathy with spike–wave activation in sleep; CAE, childhood absence epilepsy; JAE, juvenile absence epilepsy; JME, juvenile myoclonic epilepsy; GTCA, epilepsy with generalized tonic–clonic seizures alone; FS, febrile seizures; MTLE, mesial temporal lobe epilepsy.

**Figure 2:**
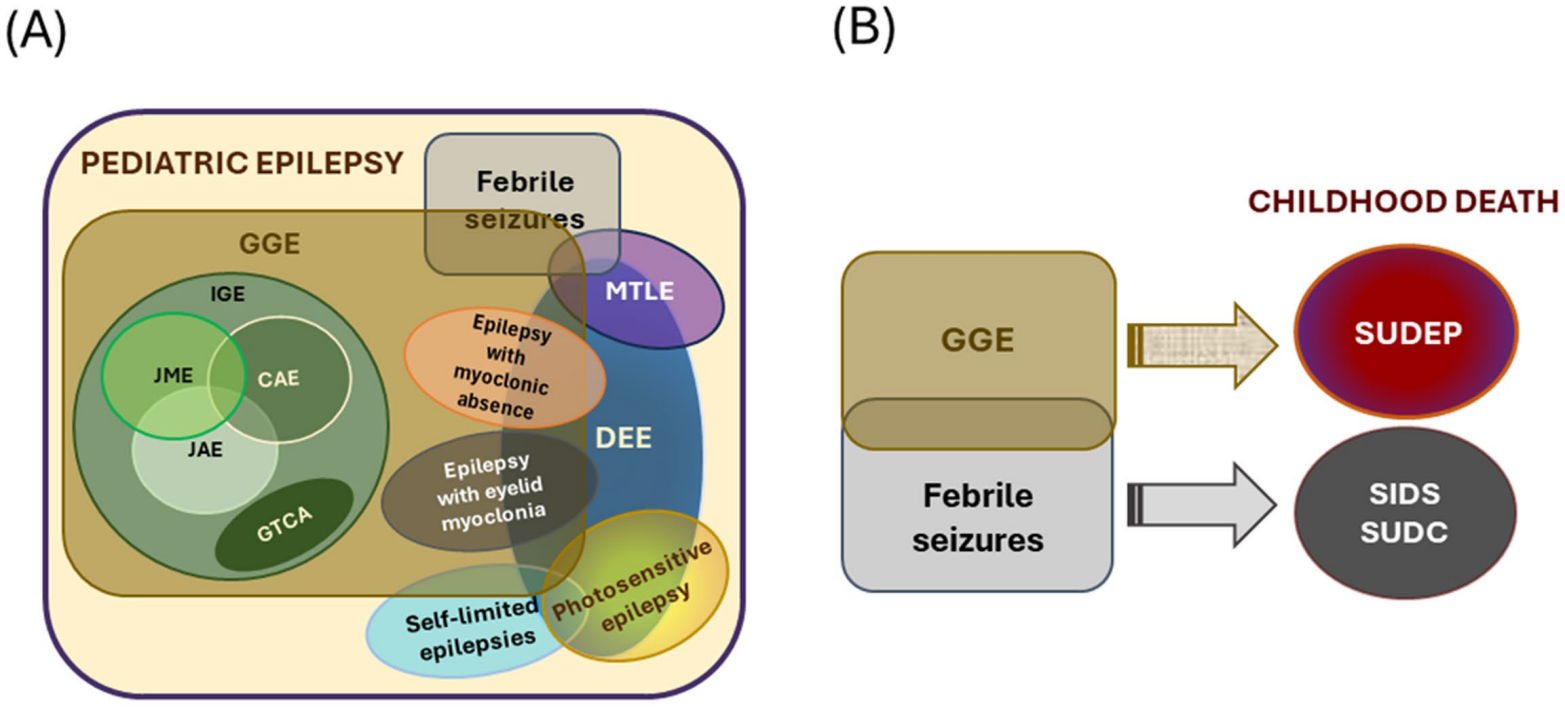
Types of pediatric epilepsies and connection to childhood death. **(A)** Schematic demonstrating overlapping features of different types of early-onset epilepsy. The overlapping sets mark the complexity of the scenario. **(B)** Genetic generalized epilepsies (GGE) and febrile seizures often make children predisposed to various premature death. The size of the circles/ovals is arbitrary and does not indicate the frequency of occurrence or other parameters. IGE, idiopathic generalized epilepsy; JME, juvenile myoclonic epilepsy; CAE, childhood absence epilepsy; JAE, juvenile absence epilepsy; GTCA, epilepsy with generalized tonic–clonic seizures alone; MTLE, mesial temporal lobe epilepsy; DEE, developmental and/or epileptic encephalopathy.

Pediatric epilepsy, defined in this review as those with seizure onset before 18 years of age, is generally classified into different types, mostly based on the age of seizure onset, set of comorbidities, and known etiologies (Figure 1). While there is significant progress in classifying pediatric epilepsy, certain limitations still exist. One of the persistent challenges is to define the boundaries of epilepsy syndromes. Multiple overlaps across categories and one type leading to or influencing the predisposition to other types of late-onset epilepsy are evident from clinical scenarios. For example, self-limited epilepsy is one of the most common and earliest types of epilepsy, accounting for about 25% of all pediatric epilepsy (22). Seizures are mostly focal in origin and subside within a few weeks or a few years after commencement, hence the name ‘self-limited’. Most patients with self-limited epilepsy respond to medication; seizures usually resolve by puberty but can occasionally occur up to 18 years of age. Interestingly, a proportion of these patients show a higher risk of developing developmental and epileptic encephalopathy (DEE) or genetic generalized epilepsy (GGE) (23–27). DEE is a broad umbrella term comprising many severe epileptic syndromes in children, each with its characteristic age of onset. DEE patients are characterized by the presence of developmental and cognitive impairment; both seizures and underlying etiology are suggested to contribute to these issues (28, 29). On the other hand, GGE is a broad term used for epilepsies with generalized seizures and genetic etiology identified through familial and twin studies (30). It constitutes 20–40% of all pediatric epilepsies (31). Considering the associated comorbidities and underlying genetic causes, the boundary between DEE and GGE often becomes diffused. Even the sub-syndromes within GGE (such as absence epilepsy and juvenile myoclonic epilepsy) show extensive overlap in terms of etiology despite having distinct electroclinical features. These overlapping phenomena make us hypothesize that different types of pediatric epilepsy generate a continuum of the disorder, considering the age of the patients, disease progression, and underlying etiology (Figure 2A).

## 4 Current challenges in pediatric epilepsy

Apart from the diagnostic challenge due to diffused overlapping boundaries across different categories, one of the most critical challenges in the field of pediatric epilepsy is to combat drug-resistance. Drug-resistant epilepsy (DRE) is defined as “failure of adequate trials of two tolerated, appropriately chosen and used antiepileptic drug schedules (whether as monotherapies or in combination) to achieve sustained seizure freedom” (32). A large proportion of children suffering from early-onset epilepsy are non-responsive to current broad-spectrum medications that are largely strategized on certain channel proteins (11, 12). Surgical removal of dysplastic tissue is currently the only treatment against recurrent seizures in these patients (33). However, considering the complexity and exceptions, the chance of developing other epileptic foci in the future is quite high, thus triggering a vicious cycle of failure and recurrence of brain surgeries in these children (34). Parallelly, long-term anti-seizure drug therapy may negatively affect their cognitive development and increase the risk of premature death (35, 36). We elaborate on some of these clinical and mechanistic challenges in the following subsections.

### 4.1 Comorbidity with neurodevelopmental disorders

Up to 40% of cases of intractable pediatric epilepsy demonstrate significant association with brain malformations (32, 37). Such epilepsies mostly begin within the first year of life and are often accompanied by intellectual delay, motor impairment, and cognitive deficits (38). Magnetic resonance imaging (MRI) in such cases reveals blurring of white-grey matter demarcation, abnormal cortical thickening and folding, enlarged ventricles, and focal malformations of cortical development (MCD) (39, 40). Further, electroencephalograph (EEG) recordings demonstrate the presence of epileptiform discharges from the specific malformed or dysplastic region of the patient’s brain (41). However, it is still debatable whether brain malformation is the primary cause of epilepsy in such patients. Clinical evidence of extended epileptic foci exists beyond the dysplastic sites in the brain, suggesting that epileptogenesis can be dissociable from brain malformation (42).

Additionally, impairment of either voluntary or involuntary motor function is very common in epilepsy. Epilepsy and motor disorders can occur independently, harboring different underlying pathophysiology. However, despite having some distinct features, many motor disorders can also imitate epilepsy and vice versa (43). For instance, besides having other causative factors and comorbidities, disorders such as cerebral palsy, muscle dystonia, and ataxia show significant association with epilepsy (44). This is mostly because epilepsy affects the brain regions that control motor coordination, such as the frontal lobe, cortico-striatal connections, and basal ganglionic regions (45, 46). To add to complications, motor dysfunctions are often difficult to recognize at younger ages due to the developing state of the brain. It is seldom clear which problem precedes the other and hence, the complexity of diagnosing the etiology and eventual treatment increases in these patients.

### 4.2 Risk of mortality

Severe epileptic episodes or underlying neurological anomalies may be fatal for infants. The underlying mechanisms driving this epilepsy-related mortality have not yet been robustly explored, making this one of the most critical challenges in the field. Epilepsy that begins within the first year of life often predisposes patients to higher morbidity potential than children with late-onset epilepsy. More dramatically, children with malignant neonatal epilepsy are >12 times more likely to die than children with epilepsy onset at age ≥1 month (47). Some epilepsy syndromes affecting children at ≥1 year of age can have favorable prognoses. However, others result in medically refractory seizures, developmental delay/intellectual disability, and other neurologic handicaps (48, 49).

Sudden unexpected death in epilepsy (SUDEP) is the leading cause of mortality in children with epilepsy (50). SUDEP is defined as “a sudden, unexpected, witnessed or unwitnessed, non-traumatic, and non-drowning death in patients with epilepsy with or without evidence for a seizure, and excluding documented status epilepticus, in which postmortem examination does not reveal a structural or toxicologic cause of death” (51). Because most SUDEP incidents are unwitnessed, the exact sequence of events remains unknown. However, SUDEP nearly invariably happens after a generalized tonic–clonic (GTC) seizure (refer to Table 1) and is more common in patients with genetic epilepsies (52, 53). Additionally, patients with refractory epilepsy, early seizure-onset, intellectual disability, and male gender demonstrate increased risk (54, 55). Current pathophysiological mechanisms behind SUDEP are identified as an interplay among cardiac, respiratory, and autonomic nervous systems (56). Recent MRI studies have revealed reduced grey matter volume in the thalamus, frontal cortex, cerebellum, serotonin-producing neurons in the raphe nuclei and brainstem areas, and increased grey matter volume in the amygdala, hippocampus, cingulate areas in a significant proportion of SUDEP cases (57, 58). However, despite monitoring epileptic patients through clinical trials, the mechanism of SUDEP still remains unsubstantiated.

Beyond SUDEP, the other clinical categories leading to mortality in the pediatric population are sudden infant death syndrome (SIDS), and sudden unexplained death in childhood (SUDC). Although causation behind SIDS and SUDC is possibly multifactorial and largely unexplained, many reports suggest epilepsy as a potential contributing factor to predispose the child towards sudden death (Figure 2B).

SIDS is defined as “the sudden and unexpected death of an infant under the age of one year that remains unexplained after a thorough review of the clinical history and complete autopsy” (59). Some of the risk factors of SIDS include male gender, history of febrile seizures (FS), and preterm birth (60). Interestingly, like SUDEP and SUDC, many cases of SIDS show hippocampal abnormalities (61, 62). However, whether these abnormalities correlate to any underlying disorder or mortality in the patients is unclear. Interestingly, a significant proportion of these SIDS cases have shown underproduction of serotonin or downregulation of serotonin receptors (62–65). Some of these infants also demonstrated reduced GABA receptor binding (66). Whether these clinical features have any impact on the excitation/inhibition balance of the neurons is worth researching.

SUDC is defined as “the unexpected death of a child over 12 months of age which appears to be inexplicable even after a detailed case investigation” (67). SUDC usually occurs between 1 and 5 years of age, and it is the fifth leading cause of death in children. Unlike SUDEP, SUDC doesn’t occur after a seizure; however, patients with a history of FS have a higher vulnerability (68). A proportion of these patients also have hippocampal anomalies (69). Whilst clinical evidence suggests a relationship between hippocampal anomalies, FS history, and SUDC, the nature of this association is currently unclear.

Although there is awareness of SUDEP, SUDC, and SIDS, the etiology and mechanisms behind these sudden deaths are largely unknown. Most of the case studies are limited due to the spontaneity as well as the rarity of such cases, thus being difficult to model. A common link among SUDEP, SUDC, and SIDS could be the history of FS and that the brain regions in respiratory and circulatory regulation are involved and altered (57, 70). Indeed, abnormal breathing patterns, hypoxemia, and hypercapnia are more commonly observed, but the correlation is inconsistent (71–73).

### 4.3 Complex genetic etiology

Many genetic mutations are being identified in association with epilepsy; some important ones are listed in Table 2. Around 40–70% epilepsy cases show genetic etiology (74, 75). In certain epilepsy types, the genetic cause is well known, like *SCN1A* mutation in Dravet syndrome (28, 76). However, in most cases, the genetics are inferred based on the familial inheritance pattern. For instance, pediatric generalized epilepsies have a strong heritable nature (77), but the specific genes responsible for them are not fully known. Unfortunately, simple inheritance patterns are very rare in epilepsy. Pediatric epilepsy shows complex inheritance with varying expressivity patterns. It is often observed that mutations in the same gene can cause variable phenotypes in patients. This phenomenon is well characterized in patients with *SCN1A* mutations, which show a range of phenotypes from FS to Dravet syndrome (78). It is hypothesized that these phenotypes are dependent on mutation severity. Mild missense mutations in *SCN1A* are often identified in FS patients, while more severe loss-of-function mutations are associated with severe epilepsies such as Dravet syndrome (78, 79). Interestingly, phenotype variation is observed even in cases where the mutations are the same. For example, patients with mutations in the PI3K-AKT–MTOR pathway show a spectrum of developmental and epileptic phenotypes with varying severity (80). Mutations in genes coding for MAST (microtubule-associated serine/threonine) kinases (*MAST1-4*), upstream of the PI3K pathway, have recently been associated with developmental abnormalities, epileptic seizures, and cognitive impairment (81–83). Epilepsies related to *MAST* genes have also been categorized within DEE and GGE due to overlapping etiology, thus complicating the categorization. Conversely, there are multiple genes that are shown to be associated with the same epilepsy syndrome, as seen in the case of DEE (84–86). Further contradictions arise when genotype–phenotype cannot be correlated directly. For instance, the severity of phenotypes in patients with mutation in ciliary gene *CDKL5* does not solely depend on the primary mutation, but also on the interplay between intrinsic and extrinsic factors (87). Such cases are difficult to treat and are often intractable.

**Table 2:** Summary of different genes and environmental factors implicated in pediatric epilepsies.

Causative factor type	Causative factor	Type of epilepsy associated with	References
Signaling defects	*kRAS*	Temporal lobe epilepsy, DEE	(283, 284)
	*BRAF*	Focal/Generalized epilepsy	(285)
	*NF1*	Focal/Generalized epilepsy	(286, 287)
	*PIK3CA, AKT3, PTEN, MTOR*	Focal/Generalized epilepsy	(80, 109, 288)
	*TSC1, TSC2*	Temporal lobe epilepsy	(289, 290)
	*NPLR2, NPLR3*	Temporal lobe epilepsy	(291)
	*DEPDC5*	Temporal lobe epilepsy	(292)
Abnormal neural migration	*RELN*	Temporal lobe epilepsy	(123, 293)
	*DCX*	Temporal lobe epilepsy	(198)
	*LIS1*	Temporal lobe epilepsy	(294)
Ciliopathy	*EFHC1*	JME	(295)
	*CILK1*	JME	(223)
	*CDKL5*	JME, DEE	(296, 297)
Channelopathy	*SCN1A*	DEE, Dravet syndrome, MTLE	(76, 291, 298, 299)
	*SCN2A*	SeLNE, SeLIE, DEE, Febrile seizures	(300–302)
	*SCN8A*	SeLIE, DEE, JAE	(303–305)
	*GRIN2A*	SeLECTS	(306)
	*KCNT1*	EIMFS	(307)
	*KCNQ2, KCNQ3*	SeLNE, DEE	(308–310)
	*KCNJ10*	GGE, MTLE	(311, 312)
	*CACNA1A*	GGE, DEE	(313–315)
	*CACNA1H*	GGE	(316–318)
	*HCN1*	Febrile seizure, DEE	(319, 320)
	*GABRA1A, GABRG1*	Dravet syndrome, CAE, JME	(321–325)

SeLNE, self-limited neonatal epilepsy; SeLIE, self-limited infantile epilepsy; SeLECTS, self-limited epilepsy with centrotemporal spike; DEE, developmental and/or epileptic encephalopathy; GGE, genetic generalized epilepsy; JME, juvenile myoclonic epilepsy, JAE, juvenile absence epilepsy, EIMFS, epilepsy of infancy with migrating focal seizures; CAE, childhood absence epilepsy.

The varying expressivity of epilepsy phenotype is hypothesized to be associated with genetic modifiers (88). Genetic modifiers are gene variants or non-coding single nucleotide polymorphisms (SNPs) present in a patient, in addition to the primary mutation, that are instrumental in modifying the phenotypic outcome in that patient. So, the final phenotype is determined by the close epigenetic, genetic, or functional interactions between these variants, which breaks the dogma of epilepsy being a monogenic disease. We think that identifying such novel epilepsy modifier genes can enhance our understanding of the underlying mechanisms.

## 5 Modeling epilepsy

Despite our understanding of the underlying causes and characteristics of various epilepsy syndromes, pediatric epilepsy remains a burden on the healthcare and economic sectors, mainly due to the predominance of drug-resistance and premature deaths (8–10). This emphasizes the importance of investigating the molecular and developmental mechanisms underlying early-onset epilepsies, which can eventually aid in designing targeted therapeutic strategies. To achieve this, epilepsy models that can closely recapitulate the human specific features are necessary. An ideal model should show construct validity (recapitulation of patient-specific etiology), face validity (replication of patient-specific phenotypes), and predictive validity (responding to treatments that can be effective in humans) (89). Although epilepsy has been modeled in various non-mammalian systems, such as *Drosophila*, *C. elegans*, and zebrafish, we consider rodents as a stronger candidate for being a model organism for epilepsy and neurodevelopmental disorders. This is because mice and humans show over 99% genetic homology, and more importantly, they show similarity in many stages of neurodevelopment and can undergo genetic manipulation to introduce patient-specific mutations for testing (90, 91). Currently, several mouse models are being used to study epilepsy; herein, we discuss different types of them. Broadly the mouse models of epilepsy can be categorized as induced models and genetic models.

### 5.1 Induced epilepsy models

An induced epilepsy model is where seizures are triggered in healthy mice by chemical or electrical stimulation of the brain (92). This is amongst the oldest strategies and is used extensively in current research as well. For chemical stimulation, an intracerebral or systemic injection of some of the neuroexcitatory drugs is used. Kainic acid is one of the first compounds used to recapitulate temporal lobe epilepsy (TLE), a common kind of focal epilepsy observed in both adults and adolescents (93, 94). It is a glutamate analog, and its administration causes neuronal depolarization, particularly in the mouse hippocampal region (95). However, patients with TLE also show neurological compromise in extrahippocampal regions (96, 97). Pilocarpine is often used to produce lesions in neocortical areas along with the hippocampus (98). Other compounds like pentylenetetrazol (PTZ), strychnine, N-methyl-D, L-aspartate, and penicillin are also used as convulsants to model seizures, and loosely epilepsy (92). On the other hand, electrical stimulation involves implanting electrodes in the region of interest and stimulating them to generate seizure outcomes (99). Less invasive methods, such as whole brain stimulation via trans-auricular or trans-corneal surface electrodes, are also available (100). It is important to note that most of the above-mentioned induced models exhibit acute seizures. Chronic seizures can be induced by kindling wherein mice exhibit spontaneous seizures after repeated electrical or chemical stimulation (101). Nevertheless, induced models generally do not validate the actual etiology of the disorder, especially with respect to a large proportion of pediatric epilepsy patients. In this regard, genetic models emerge as a more realistic tool to study underlying mechanisms.

### 5.2 Genetic epilepsy models

Advancements in gene editing technology have allowed researchers to integrate many patient-specific gain-of-function or loss-of-function genetic mutations in mice. Such models are very instrumental in understanding mechanisms as they have high construct validity. These models also provide a strong platform, allowing preclinical testing of small molecules. Some of the well-known mouse models of epilepsy include constitutive knockout of channel proteins such as *SCN1A, SCN2A, SCN8A, KCNA1, GABRA1, KCNQ2,* and many more (102–105). These models have not only helped us to understand the mechanistic aspect of these mutations but also the associated behavioral and developmental consequences. Parallelly, signaling pathways, especially the PI3K-AKT–MTOR pathway, are associated with pediatric epilepsies. Unlike most of the ion channels, the constitutive knockout of such pathway genes is often embryonically lethal (106–108). Furthermore, a large proportion of pediatric epilepsy patients exhibit spontaneous or *de novo* mutations in the pathway genes, where the phenotypic severity is often dependent on the developmental timing and regional extent of the mutation (109, 110). This further complicates the modeling design for such specific genetic variants since a constitutive deletion strategy may not mimic the actual scenario in such cases. To circumvent this problem, conditional strategies such as Cre-lox or FRT-Flp systems are used to induce the patient-specific mutations in specific cellular lineages, especially for genes associated with signaling pathways like PI3K-AKT–MTOR (111–115). The developmental timing of the genetic mutation can be more finely regulated by using inducible *cre* systems (116). Such strategies give more flexibility and allow researchers to spatially and temporally control their genetic modifications. Moreover, it allows partial manipulation of the genes that are important for early embryonic development and are fatal to mice if removed. While such conditional lines provide cell lineage and variant-specific modifications of the gene of interest, even finer focal genetic perturbations are possible in mice. One such example is a *Pten* mouse model, wherein an exogenous viral vector carrying Cre has been injected into the hippocampal area in *Pten* floxed background (117). By controlling the dose and injection site of the viral vector, one can create a mosaic loss of *Pten* in the region of interest. Another approach is to perform *in utero* electroporation (IUE), where a plasmid DNA targeting the gene of interest is injected *in utero* into the embryonic brain ventricles and then electroporated to the region of interest using a pair of electrodes. IUE strategy is widely implicated in inducing activating PI3K-AKT–MTOR pathway mutations in rodent systems (118–120). IUE strategy provides a deeper understanding of the function of a particular gene in a specific cellular population. These models are very relevant in studying developmental mechanisms underlying MCD-associated epilepsies or focal epilepsies, wherein patients often show somatic mosaicism (110, 121).

## 6 Mechanisms underlying epilepsy

Much of our current mechanistic understanding of pediatric epilepsy has come from studying model systems (mentioned in the previous section). Since the start of the epilepsy field, epilepsy has been always mechanistically attributed to mutations in ion channels and, in turn, alterations in neuronal excitability and inhibition (122). However, research over the past few decades has identified severe non-ion channel-associated genes being associated with pediatric epilepsy (109, 123–126). These studies highlighted novel mechanisms underlying epilepsy beyond ion channels. Herein we discuss some of these mechanisms.

### 6.1 Mechanisms involving ion channels: channelopathy

Several studies have associated either voltage- or ligand-gated ion channels with monogenic epilepsies, where the seizure onset coincides with the temporal expression of the affected channel during development (127–131). Mutations in ion channels are involved in different types of pediatric epilepsies, such as self-limiting epilepsies, generalized epilepsies, epileptic encephalopathies and FS (Table 2). Channel proteins and epilepsy by itself is a huge topic to cover; and has been discussed in vast lengths in several reviews and book chapters previously. In this review, we are only providing a brief overview of the mechanistic relevance of different channel mutations causing epilepsy; further elaboration is beyond the current focus of this review.

**Figure 3:**
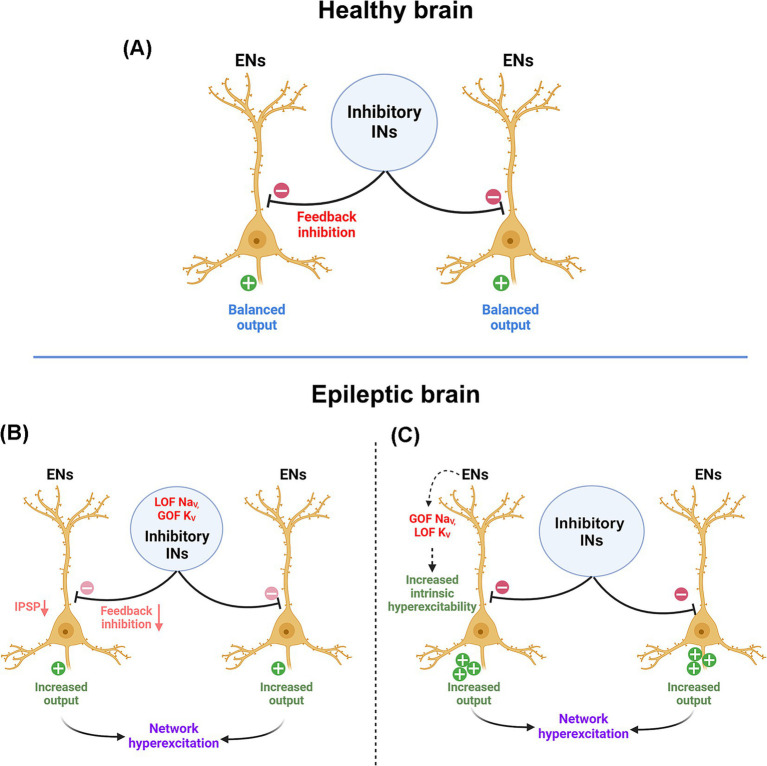
Network excitation in healthy and epileptic brain. **(A)** In a healthy brain, excitatory neurons (ENs) generate a balanced output due to feedback/feedforward inhibition from interneurons (INs). **(B)** In epileptic patients with channel mutations, this inhibition is reduced due to hypoexcitability or impaired action potential propagation in interneurons, or **(C)** ENs become inherently hyperexcitable or have a lower threshold for action potentials. These events can occur independently or together, leading to network hyperexcitation. ENs, excitatory neurons; INs, interneurons; IPSP, inhibitory postsynaptic potential; GOF, a gain of function; LOF, loss of function; Na_V_, voltage-gated sodium channel; K_V_, voltage-gated potassium channel.

Voltage-gated sodium channels (Na_V_) allow voltage-dependent influx of Na^+^ ions to initiate neuronal depolarization; different genetic mutations in these channels can affect the functioning in varied ways (132). For instance, mutations in the inactivation gate domain of Na_V_ causes the channel to close slowly or incompletely during the depolarization period, leading to an excessive influx of Na^+^ ions inside the neuron, making it intrinsically hyperexcitable (133, 134). In contrast, mutations that allow channels to recover faster from the inactivation state cause increased firing frequency. Some also lower the action potential threshold, causing neuronal hyperstimulation (133). Na_V_ loss-of-function mutations found in inhibitory interneurons, in turn, can result in disinhibition in neural networks (Figure 3) (135). Again, voltage-gated potassium channels (K_V_) are critical for neuronal repolarization and bringing the membrane to the resting state. K_V_ mutations can alter the electromechanical coupling in the channel, preventing it from sensing voltage (136); this results in repetitive neuronal firing and altering excitation/inhibition balance. Apart from the propagation of action potential, voltage-gated ion channels, such as voltage-gated calcium channels, trigger neurotransmitter release from presynaptic boutons. Genetic variants in a family of voltage-gated calcium channels are also associated with epilepsy (137, 138).

Ligand-gated ion channels are typically found on the postsynaptic membranes or dendritic spines and play a vital role in signal reception. Mutations in these channels alter the ligand binding affinity and are implicated in epileptogenesis (139). For instance, mutant acetylcholine receptors get activated and remain open at abnormally lower levels of acetylcholine, causing hyperexcitability (140). Some mutations also impair the assembly or reduce current flow through ion channels, potentially increasing the net microcircuit excitability (141, 142). Not all mutations lead to altered biophysical properties of a channel. Some biophysically silent mutations keep the structural configuration undisturbed. However, their subcellular localization, expression levels, or affinity to the cytoplasmic interactor proteins may alter, disrupting membrane properties. Indeed, this feature is very well characterized in NMDA and AMPA receptors, which play important roles in long-term potentiation (LTP) and long-term depression (LTD) (143, 144). Patients harboring mutations in these receptor genes develop seizures, along with developmental and cognitive comorbidities (145–147). Similarly, there are molecules which aid in physical connection of synapses, primarily belonging to the neurexin-neuroligin family. Mutations in both have been associated with epilepsy (148, 149). Expression of neurexin-neuroligin proteins was reported to be increased in epileptic patients (150). Interestingly, this family of proteins was originally associated with neuropsychiatric conditions like autism spectrum disorder (ASD) (151). However, its newly identified role in epilepsy may provide insights into uncovering shared mechanisms between epilepsy and ASD.

Most of these mechanistic insights gained impetus from studying structural-biochemical properties and electrophysiological features of the channel proteins in various model systems, such as cell lines, oocytes expressing channel variants, and artificial modeling which are far away from the actual human condition (141, 142, 152). Unfortunately, studying isolated molecules or cells does not reveal the full picture, considering epilepsy is a network phenomenon. Another major caveat was that most channel-related research has an extreme bias toward neuronal activity, neglecting the potential roles of other neural cell types. Research over the past few decades has proven the importance of glia in modulating synaptic modulation and transmission by uptaking/redistributing ions, glucose, and water molecules at the synapse (153–155). In fact, patients with mesial temporal lobe epilepsy (MTLE) often carry mutations in inwardly rectifying potassium channels (*KCNJ10*) and water channels (*AQP4*), that are abundantly expressed in astrocytes (156–158). Our understanding of neuron-astrocyte interaction and its association with epilepsy currently remains limited. However, this association is gaining more and more relevance toward developing novel anti-epilepsy strategies.

### 6.2 Mechanisms involving proliferation and maturation of neural cells

**Figure 4:**
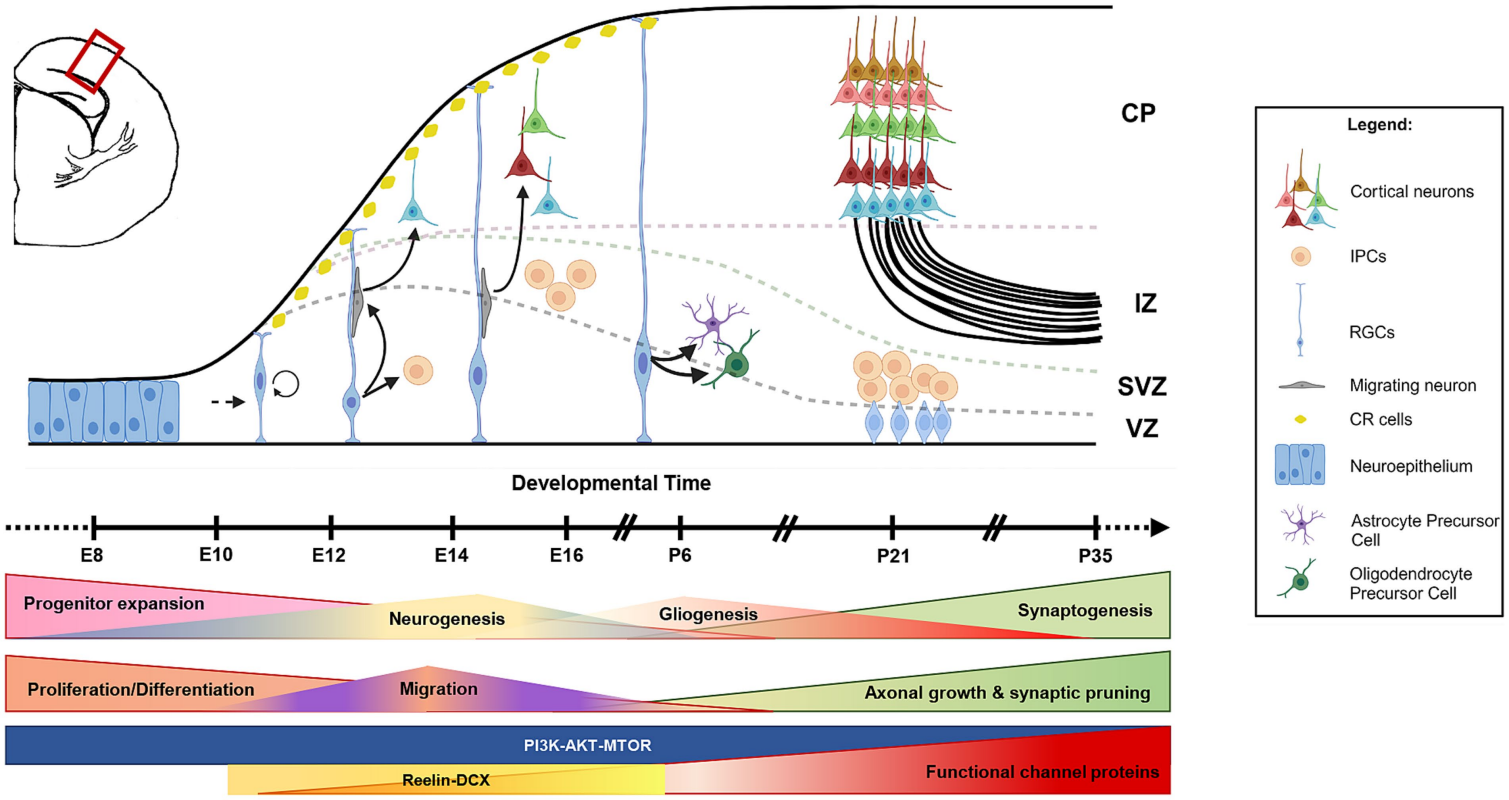
Cascade of neurogenesis and gliogenesis in developing mouse brain. The schematic of a coronal hemi-section of a developing mouse cortex shows that the initial phase of brain development involves the expansion of the progenitor pool (neuroepithelial cells and RGCs). This is followed by the formation of neurons (neurogenesis) and glia (gliogenesis) either directly (from RGCs) or indirectly (from IPCs). Newly formed neurons migrate toward the pial surface with the help of radial glial projections and occupy in an inside-out fashion (late-born neurons occupy upper layers, and early-born neurons occupy deep layers). CR cells regulate this radial migration process. Neurogenesis and gliogenesis are followed by synaptogenesis and functional network formation. RGCs, radial glial cells; IPCs, intermediate progenitor cells; CR cells, Cajal–Retzius cells; CP, cortical plate; IZ, intermediate zone; SVZ, subventricular zone; VZ, ventricular zone; E, embryonic; P, postnatal.

Besides channelopathies, multiple gene variants in critical signaling pathways, specifically PI3K-AKT–MTOR and RAS–RAF–ERK pathways, have been identified as epileptogenic (159, 160). These pathways are highly conserved across evolution and interact with each other to promote critical processes like cell growth, proliferation, differentiation, and apoptosis, as well as the generation of different neural cell types and synapses (161–168) (Figure 4). Mutations in one such pathway often impair the regulation of other pathways, causing variable consequences downstream. Since global homozygous deletion in the components of PI3K-AKT–MTOR and RAS–RAF–ERK pathways mostly caused embryonic lethality in animal models, recent studies have used brain-specific conditional genetic deletion via either *in utero* electroporation or recombination techniques to study the effects. Genetic null mutants of *Depdc5, Pten, NF1, Tsc1,* and *Tsc2* and *in utero* electroporation models for *Akt, Kras, Braf*, and *Rheb* recapitulate the range of clinical phenotypes, either fully or partially (119, 169–176). Here, we emphasize a few of these models which aided in understanding the mechanism behind pediatric epilepsy. *Pten* is one of the negative regulators of the PI3K-AKT–MTOR pathway, and its selective removal from the murine hippocampus resulted in spontaneous seizures (177). This model has also shown increased activation of the MTOR pathway. Further, gain-of-function mutations in PI3K or loss-of-function mutations in TSC1/TSC2 in the initial stages of brain development (radial glial cells, or RGCs) also resulted in seizures in the early stages of life (111, 178–180). Many chemically induced epilepsy models also show alteration in the PI3K-AKT–MTOR pathway (181). Mechanistically, *Pten, Pik3ca, Tsc1, and Tsc2* mouse models have demonstrated that pathway hyperactivation results in increased cell proliferation, cellular hypertrophy, dendritic hypertrophy, and aberrant axonal growth (111, 171, 173, 182). Besides these cell-autonomous changes, the affected cell influences the development and function of the adjacent non-mutated cells (118, 183). Similar mechanisms have been identified in mouse models harboring mutations in the RAS pathway (176, 184). We hypothesize that the mutations in these critical signaling pathways not only alter the synaptic properties but also alter the numbers and diversity of the excitatory/inhibitory neurons in the neural network, leading to network hyperexcitability. Moreover, these mutants also modeled the coexisting MCDs such as megalencephaly, hydrocephalus, and hippocampal/cortical dysplasia along with epilepsy, thus accurately mimicking the clinical scenario (111, 171, 173). This strongly suggests that disruption of neurodevelopmental processes is central to both MCDs and epileptogenesis. Besides cell growth and proliferation, MTOR activity critically maintains cellular autophagy and vesicular trafficking, thus, in turn, regulating neurotransmitter release, synaptic recycling, and mitochondrial homeostasis (185). Indeed, reduced cellular autophagy is reported in conditional knockout models for *Pten* and *Tsc1* (186). However, it remains disputed whether it is a cause or an effect (187, 188). Translation and surface expression of many ion channels are also dynamically regulated by PI3K-AKT–MTOR and RAS–RAF–ERK pathways (189). Pathway misregulation can alter the surface expression of these proteins and, in turn, cellular excitability, making this a plausible explanation for connecting signaling pathways to epilepsy.

The above-mentioned mechanisms suggest that suppression of this pathway using MTOR inhibitors should rescue the phenotype. Unfortunately, use of inhibitors such as rapamycin and its analogs showed limited success in curbing epilepsy in both mouse models as well as patients (190, 191). An instrumental study using the clinically relevant *Pik3ca* genetic models demonstrated that activating *Pik3ca* mutation causes intrinsic neuronal hyperactivity in mice, which is, in turn, acutely suppressible by PI3K and/or AKT but not MTOR inhibition (192). These findings suggest that MTOR hyperactivation is not always the sole direct reason behind these epilepsies and possibly explain the reason behind the partial or complete failure of rapamycin analogs and other MTOR inhibitors in treating epilepsies of all kinds. The mouse models also revealed that the underlying mechanism behind the emergence of epilepsy in such pathway-related mutations traces back to abnormal neurogenesis and gliogenesis (111, 159, 174, 175, 192). This re-emphasizes the importance of early developmental processes in determining brain circuitry and expands epileptic mechanisms beyond channelopathies. As we will see in the upcoming sections different components of these pathways are involved in different intracellular signaling beyond MTOR-driven cell growth.

### 6.3 Mechanisms involving neural migration

Neural migration to specific zonal layers of the brain is crucial for making appropriate axonal connections and synaptic maturation (Figure 4). Many genes are involved in regulating these processes, such as *RELN, DCX, LIS1, ARX, TUB1A,* and *FLNA*. Mutations in these migration-related genes often result in lamination defects and epilepsy in humans (193–197). Here, we focus on reelin (RELN) and doublecortin (DCX), whose functions with respect to neural migration and epilepsy are more elucidated. DCX is an X-linked microtubule-associated protein; patients with *DCX* mutations have structural cortical malformations, often associated with epilepsy (194). Specifically, these patients are mosaic for *DCX* mutations, such that a proportion of neurons migrate successfully in the cortex, while the mutant ones fail and accumulate in subcortical regions (193, 198). This abnormal neuronal localization impacts the formation of functional networks and consequently leads to epileptogenesis. Interestingly, studies on resected human brain samples demonstrated fewer Reelin^+^ and DCX^+^ cells in MTLE-hippocampal sclerosis patients and chemically-induced seizure models (199–202). Rodent models of clinically relevant *DCX* mutations displayed impaired migration of hippocampal granule cells and spontaneous seizures (203, 204). A double-null model for *DCX* and *Doublecortin-like kinase 1* was epileptic and exhibited more severe migration abnormalities in cortical projection neurons and inhibitory interneurons (205, 206). Similarly, Reelin is involved in neural migration, especially during the early period of embryonic neurogenesis and hippocampus formation. At this stage, Reelin is expressed in Cajal–Retzius cells that play instrumental roles in the inside-out layer formation of the neocortex as well as in hippocampal lamination. Indeed, focal malformations in cortex and hippocampal lamination defects are observed in patients with *RELN* mutations (207, 208). These patients also exhibit intractable epilepsy (209). Similar phenotypes were also observed in the *Reeler* mouse mutant (210). Later in development, reelin is also expressed in inhibitory GABAergic interneurons, which are important for regulating network excitability as well as synaptic maturation of hippocampal granule cells (211–213). Reduction in the interneuron number due to *RELN* mutation is hypothesized to cause network hyperexcitability (202, 214). However, this hypothesis is debatable as many clinical reports lack proper age-matched controls. In a recent retrospective pediatric brain study, no change was observed in the Reelin^+^ cell number in the hippocampus of epileptic human brains as compared to that of age-matched controls (215). Moreover, variability in immunohistochemistry results is common due to differential post-processing time for human brains (216, 217). On the other hand, commonly used rodent models, like kainic acid-induced rat seizure model, are complex to analyze; kainic acid itself induces death of hippocampal neurons. To complicate further, Reelin is known to be an upstream regulator of PI3K and RAS pathways (218). So, alteration in the reelin expression may have consequences with respect to cell proliferation and maturation; but these associations are yet to be proven. Taking together, it remains to be determined whether neuronal migration defects directly lead to epilepsy.

### 6.4 Mechanisms involving genesis and function of cilia: ciliopathy

**Figure 5:**
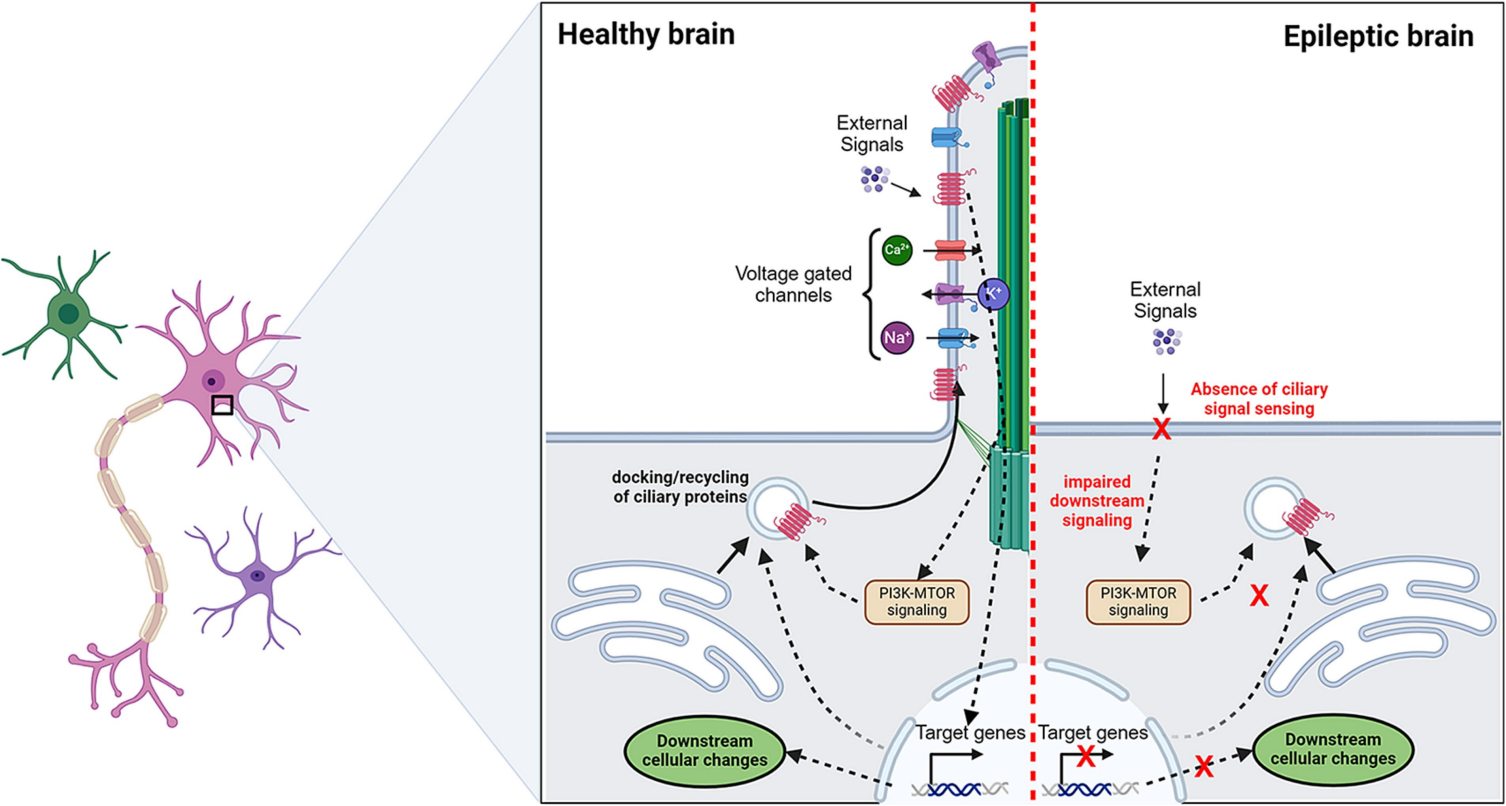
Potential role of primary cilia in epilepsy. Primary cilia sense the presence of extracellular cues via signaling receptors. This leads to a cellular cascade that may lead to activation/inactivation of a set of genes. These transcriptional changes can alter the docking or recycling of surface proteins which include signaling receptors and ion channels. In epilepsy, the absence of primary cilia is observed in a proportion of cells. This makes cells insensitive to “cilia-mediated signal sensing.” Making aberrant regulation of ion channels which may tweak the excitation/inhibition balance. Dotted arrows indicate the proposed pathways.

Ciliary genes have recently been associated with epilepsy. There are two types of cilia present in the brain: primary/nonmotile cilia and secondary/motile cilia. Primary cilium is a specialized organelle found in almost all neural cells that senses and reacts to most of the signals and external environmental cues (219, 220). These cilia are rich in signaling receptors and ion channels, which are essential for brain growth and function (219). Therefore, it is no surprise that mutations in the genes involved in ciliary maturation and function cause neurodevelopmental disorders including epilepsy. Clinical studies have reported that a significant proportion of patients with juvenile myoclonic epilepsy (JME) harbor mutations in the genes involved in primary cilia formation, like *CILK1, EFHC1,* and *CDKL5*, while reduced number of primary cilia was identified in surgically resected brain samples of focal cortical dysplasia (FCD) patients (220–225). Such clinical findings hint at the role of primary cilium in epileptogenesis (Figure 5). However, the precise mechanistic relation between primary cilia dysfunction and epilepsy is yet to be known. Animal models indicate the importance of primary cilium in neural cell proliferation, differentiation, migration, and synaptogenesis (226, 227). Considering that primary cilia are like signal sensors for a cell, their loss possibly makes neurons insensitive to external cues such as neuromodulators and causes epilepsy (Figure 5). However, a few contradictory reports challenge this hypothesis. A mouse model harboring a patient-specific variant in *CILK1* was epileptogenic, while another study on that identical variant failed to identify any epileptic behavior in mice (223, 228). Such contradictory results prevent us from confirming the direct correlation between ciliopathies and epilepsy. Nevertheless, the field of primary cilia is gaining momentum in the field of epilepsy. Very recent high-resolution electron microscopy data from human brain slices have shown that primary cilia are diverse in their shape, size, and microtubule architecture depending on the cell type and brain regions, which in turn can diversify the signaling competencies (229). This structural range of primary cilia provides each neuron or glial cell with a unique barcode of access to the surrounding neural network which influences the overall network excitability. Hence, primary cilia have now been considered an integral component of the synaptic signaling and neural connectome.

Even less information is known about motile cilia dysfunction and epilepsy. Motile cilia, present on brain ependymal cells, play an integral role in the circulation of cerebrospinal fluid. Recent studies indirectly suggest that blockage of fluid flow underlies epilepsy development (19, 230). This disrupted flow of cerebrospinal fluid is attributed to impaired development and function of ependymal cilia. However, the ciliopathy field is still in its infancy due to the cilium’s high structural complexity and diverse functional range based on cell type and developmental stage. Interestingly, the PI3K-AKT–MTOR pathway is very central to this process as well. Crosstalk between the PI3K-AKT–MTOR pathway and cilia is evident, with defects in the pathway function having adverse effects on the cilium length or even the development of ependymal cells (231–233). However, no direct role of MTOR-related ciliopathy in epilepsy has yet been established. Nonetheless, it opens a new avenue for understanding epilepsy and related neurodevelopmental disorders.

### 6.5 Epilepsy and association with sleep and body clock

**Figure 6:**
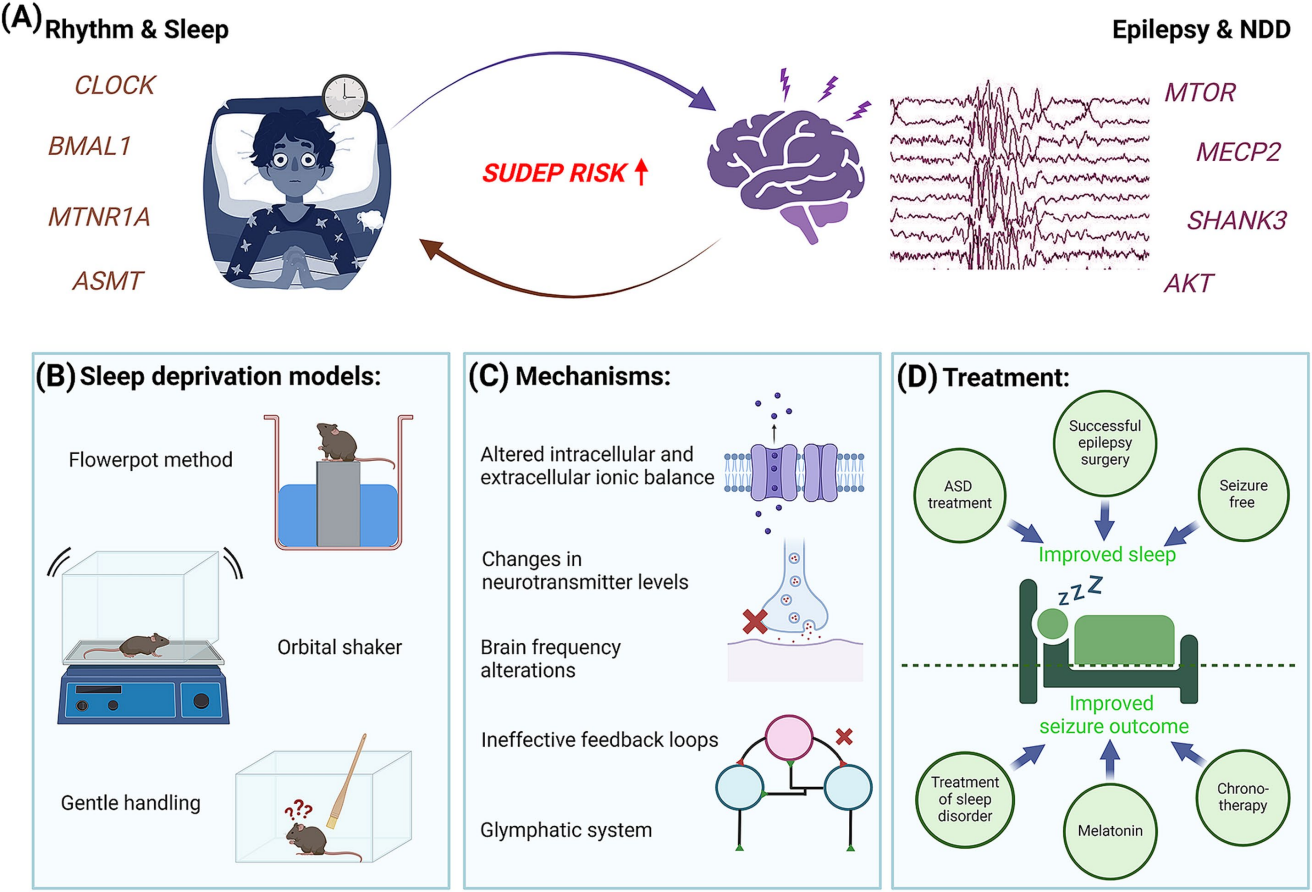
Interconnection of epilepsy with body rhythms and sleep. **(A)** Summary diagram representing the close association between sleep deprivation and epilepsy. Schematic demonstrates a patient suffering from either sleep deprivation or epilepsy triggers a higher risk of SUDEP. **(B)** Sleep deprivation in rodents is induced by changing the environment or application of stress. **(C)** Some of the common molecular and physiological mechanisms underlying sleep deprivation and epilepsy are shown. **(D)** Close association of sleep deprivation and epilepsy introduces a novel therapeutic angle.

Beyond direct mechanisms through ion channels or neurotransmitters, novel anti-epilepsy therapies can also be developed by studying body homeostasis and body cycles, including sleep and wakefulness. Epilepsy and sleep have been bidirectionally associated with each other for centuries (Figure 6). In other words, epilepsy can cause sleep problems in patients while sleep deprivation may trigger certain types of epilepsy (234, 235). Especially children with epilepsy have been documented for poor sleep quality, increased nocturnal awakenings, early morning awakenings, difficulty in falling asleep, and/or excessive daytime sleepiness. Certain epileptic seizures occur consistently during specific stages of sleep–wake cycles, suggesting a strong correlation between the two. Even with respect to the risk of mortality, most cases of SUDEP and SIDS occur during sleep, with SIDS specifically linked to the rapid eye movement (REM) sleep stage (56, 236, 237). In other cases, increased synchronous neuronal firing during non-REM (NREM) sleep may make the brain more susceptible to seizure activity, while inhibition of thalamocortical synchrony during REM lowers the epileptiform brain activities (238, 239). This association is often taken as an advantage to diagnose seizure activities. For instance, sleep deprivation is used before EEG recording, both in epilepsy models and pediatric patients, to evoke heightened neuronal activity (111, 240).

The underlying reason behind this interesting connection may be aligned to changes in neurotransmitter levels, intracellular and extracellular ionic balance, alterations in the duration of sleep stages and in brain wave frequencies, ineffective feedback loops, and other possibilities (241, 242). Some reports also link sleep disruption and pediatric epilepsy through stress and induced neuroinflammation, marked by altered expression of inflammatory mediators and glial activation (243, 244). Further, the glymphatic system, a recently discovered waste clearance system of the central nervous system, is being considered as a potential mechanism connecting sleep and many neurological disorders including epilepsy, such that its dysfunction may account for the common association between disturbed sleep or sleep deprivation and increased seizure risk (245–247). This phenomenon, in turn, can critically disrupt normal brain development and cognition, more prominently if it begins at early stages in neonatal and pediatric populations (241). Although recent studies are trying to understand the effect of current anti-seizure drugs on the glymphatic system (248), there are a lot of open questions that remain unaddressed.

Curbing sleep issues is proving a good way to attenuate the severity of seizure episodes in children, thus becoming potential avenues for anti-epilepsy therapies. Certain anti-epileptic drugs have analgesic effects that help to alleviate sleep quality in some pediatric patients. Administration of melatonin, an endogenous hormone that inhibits brain excitability and induces balance in sleep–wake cycles and circadian rhythms, is found to be neuroprotective and anti-convulsant in nature in both patients and mouse seizure models (242, 249–251). The mechanism is based on the activation of two high-affinity G protein-coupled melatonin receptors, MT1 and MT2, which provide beneficial effects on regulating the circadian and sleep pathways without the baggage of side effects seen due to regular use of sleep medications (252). Slow-release melatonin and its agonists are currently being considered as sources of novel, efficacious therapeutics against certain types of epilepsies and sleep disorders (242, 252, 253). Parallelly, core circadian proteins, BMAL1 and CLOCK, have been shown to influence excitability and seizure threshold by regulating the PI3K-AKT–MTOR pathway (243, 254–257). Studies also reveal a feedback loop where MTOR activates BMAL1 via phosphorylation (254). Epilepsies associated with PI3K-AKT–MTOR pathway-related brain malformations also show a dysregulation of the expression of core circadian genes, thus making them excellent therapeutic targets for treating this specific type of epilepsies (258, 259). Consequently, the therapeutic possibilities for optogenetics and chronotherapy are actively being considered to treat seizures, especially those that are known to have a circadian sleep component as an underlying mechanism. Finally, given the considerable proportion of DRE in children, we feel an obvious treatment strategy would be toward advancements in personalized anti-epileptic treatment paradigms, using the time-dependent information relevant to the individual patient. This information may include times of day that witness the greatest occurrence of seizures of the highest levels of epileptogenicity in relation to sleep, wakefulness, and other body rhythms (242, 260).

## 7 Limitations of epilepsy models and novel advancements

Despite using diverse model systems, researchers continue to face challenges ranging from proper disease recapitulation to discovering novel therapeutics. The aforementioned induced epilepsy models are currently used by large screening consortia, such as the Epilepsy Therapy Screening Program, due to the ease and high throughput capacity (261). However, some of these inducible agents are known to cause neuronal loss and behavioral changes in mice. Moreover, chemical or electric stimulation is not a true cause of epilepsy in patients, restricting our capacity to extrapolate the findings in a human context. Conversely, genetic models recapitulate the genetic etiology and physiology of epilepsy to a much greater extent. Unfortunately, these models also face major challenges. One such challenge is the uneven frequency of spontaneous seizures, causing difficulty in assessing the output. The seizure onset in such models can also be different from that of actual patients. Besides seizure rarity, many models also exhibit absence or non-convulsive seizures, which are generally difficult to detect. In contrast to the models with low seizure frequency, there are models that suffer from severe and fatal seizures. These animals die after one or a few seizures, making them not useful for long-term epilepsy research, but may be beneficial for understanding SUDEP. Finally, genetic models face serious challenges to recapitulate the varying expressivity of epilepsy as seen in patients. As mentioned in Section 4, mutations in the same gene can cause a range of phenotypes in patients. This signifies the need to study gene interactors and modifiers in model systems. Recently, attempts have been made to investigate these connections by introducing the same mutation into mice of different strains (262–264). These studies have reported that the onset and frequency of seizures vary depending on the inbred strain used. We consider the introduction of both primary and modifier mutations in models as a useful alternative strategy to mitigate the challenge and advance our understanding of genetic modifiers.

Further, rodents cannot recapitulate certain genetic and developmental traits unique to humans regardless of the similarities (265). Due to this, not all aspects of human epilepsy can be accurately modeled in rodents, leading to unsuccessful preclinical trials. To overcome this, researchers have recently started using patient-derived induced pluripotent stem cells (iPSCs), brain organoids, and assembloids to generate disease-associated cell types for understanding the pathophysiology of certain diseases (266, 267). However, genetic variability among different iPSC lines and failure in developing the complete brain still limit the usefulness of such models (268, 269). Combining *in vivo* rodent models and *in vitro* human models seems a more efficient strategy to shed light on epilepsy. Recently, human-mouse chimeric brain models, or humanized mice, have been generated wherein patient-derived iPSCs are engrafted in different regions of the brain (270). This allows the integration of human neural cell development and function in vivo, thus aiding the enhancement of our understanding of human brain development and epilepsy.

In addition to the limitation of having accurate model systems, a major challenge in epilepsy management is pharmacoresistance. Despite proper diagnosis and drug treatment, seizures often tend to stay unresolved. Unfortunately, little is known about what causes this drug resistance. Alteration of the blood–brain barrier or drug targets due to SNPs, environmental influence, genetic background, and associated comorbidities are some of the hypothetical mechanisms underlying DRE (271, 272); however, nothing is yet proven preclinically or clinically (273). Some novel therapeutic approaches, including vagus nerve stimulation (VNS), responsive stimulation, and deep brain stimulation, involve invasive surgical intervention (274–276). Although these strategies have shown some success in recent times, their invasive nature limits their application in pediatric patients. On the other hand, an increase in extracellular serotonin (5-HT) levels is reported to inhibit various seizure types (277). Complementarily, patients with genetic or acquired 5-HT defects are more susceptible to SUDEP (278). In fact, stimulation of 5-HT receptors beneficially influences certain preclinical epilepsy models (279). Clinically, rapamycin and its analogs are currently the only pathway-related medications utilized to treat epilepsy noninvasively (280, 281). Unfortunately, these drugs work on a specific cohort of tuberous sclerosis patients, harboring rare mutations in *TSC1/TSC2,* resulting in overactivating the PI3K-AKT–MTOR pathway (159, 282). Recently, a preclinical study has shown that targeting different upstream components of the PI3K-AKT–MTOR pathway but not MTOR can acutely treat epilepsy, suggesting that epilepsy is more than channelopathies and TORopathies (192). This study also suggested that developing epilepsy may have distinct acute and chronic mechanisms that can differentially respond to the administered cocktail of anti-seizure medications. Together, such studies highlight the need to further explore different mechanisms underlying pediatric epilepsy and to develop a patient-specific therapeutic approach.

## 8 Discussion

In this review, we have discussed established and evolving cellular and molecular mechanisms underlying the development of pediatric epilepsy, emphasizing the use of diverse types of models and their pros and cons. Perspectives regarding potential reasons behind refractory epilepsy, along with challenges and contradictions in the field, are also brought forward.

Novel epilepsy-related gene variants are progressively being identified. However, lack of adequate sample size, heterogeneity in the cohort, diagnostic biases, and differential genetic and geographical backgrounds often lead to confusion in understanding the disease manifestation across patient groups. For a long time, the cause of epilepsy was considered monogenic, primarily based on defects in single ion channel proteins. However, it has become evident that epilepsy is not merely a channelopathy. Instead, it is multigenic, often involving molecules instrumental in cell cycle and signaling, ciliogenesis, and biological rhythms. Among all epilepsies, we opine that pediatric epilepsies are possibly the most difficult ones to interpret and treat for different reasons. First, a child may suffer from multiple overlapping epilepsy types, either simultaneously or sequentially, as they age. Next, pediatric patients often suffer from one or more developmental comorbidities; this may confuse the diagnoses as the boundary between cause and effect becomes blurred in such cases. Further, causative *de novo* point mutations can be difficult to identify in a newborn. Moreover, the same gene can be associated with different types of epilepsy with variable onsets. Worst of all, pediatric patients with very early-onset epilepsies are non-verbal, extremely mobile, and cannot emote what they are experiencing, resulting in added complications and delayed detection of the disorder. Parallelly, invasive brain surgeries on a few weeks-to-month-old patients are life-threatening and painful. In such situations, recent advancements in EEG, high-resolution brain imaging, genetic screening, and detection strategies provide hope. Our review has highlighted the evolving complexity of pediatric epilepsy, its close connection with neurodevelopment and cognition, as well as the associated challenges in the field. We also put forward the concept that pediatric epilepsy is a part of the neurodevelopmental disorder continuum.

**Figure 7:**
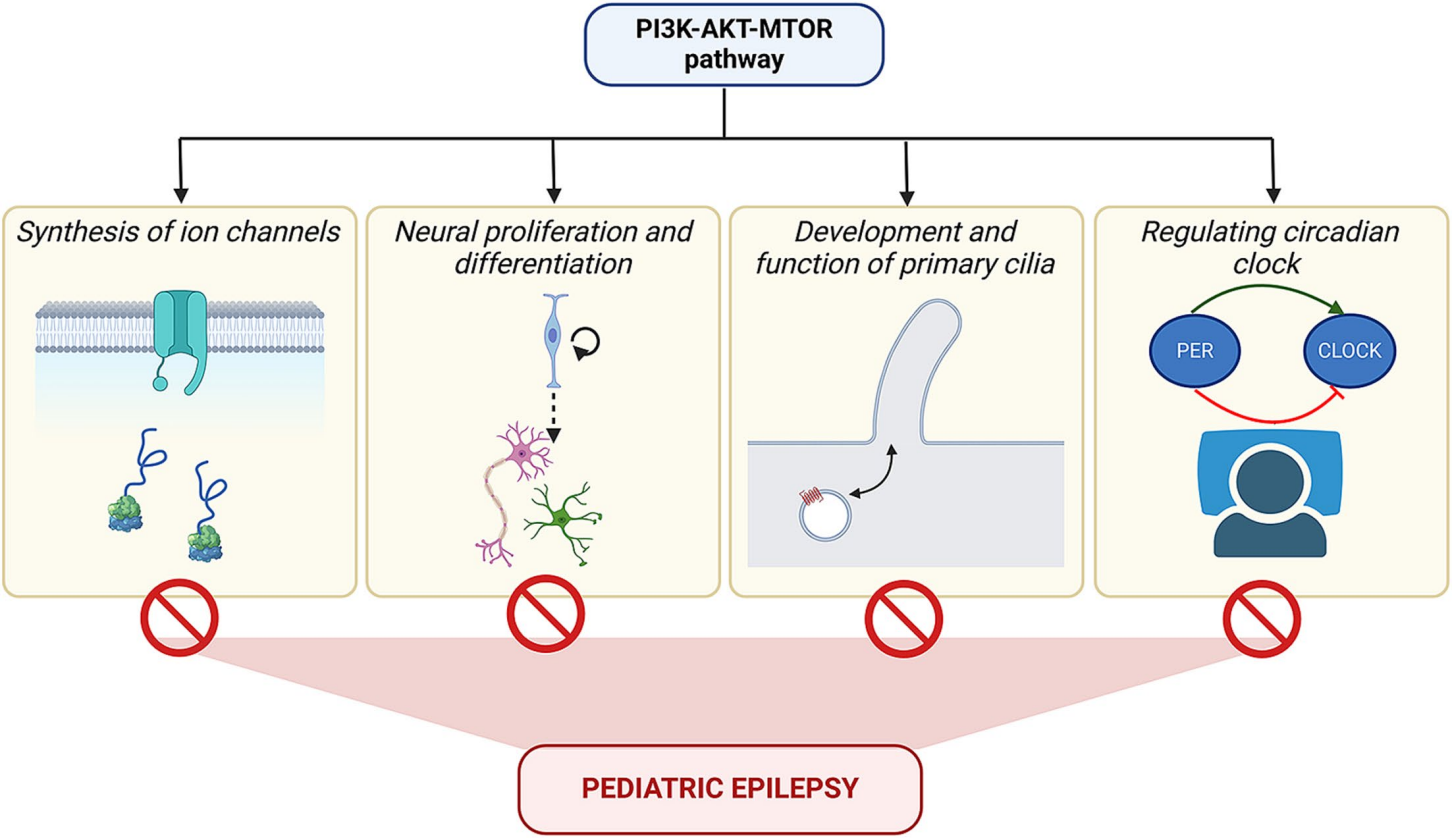
Converging influence of PI3K-AKT–MTOR pathway on epilepsy. The figure summarizes the varied roles of the PI3K-AKT–MTOR pathway in regulating channel protein synthesis, cell migration, formation of primary cilia, and molecular circuit of the circadian clock, besides its function in cell proliferation and differentiation. Mutations in the pathway components can alter these above-mentioned processes, which, in turn, may lead to epilepsy.

Finally, it is interesting to note that there is a deep underlying connection between epileptogenesis and the PI3K-AKT–MTOR signaling pathway (Figure 7). Be it the cause or the effect, the development of epilepsy is always correlated to the overactivated PI3K-AKT–MTOR pathway. As mentioned in different sections of the review, this signaling is responsible for various developmental processes, such as proliferation, neural differentiation, and formation of ion channels and cilia, as well as helps in the regulation of the circadian body clock and sleep rhythms. Disruption of any of these cellular processes can also perturb the pathway functioning as a feedback mechanism, further triggering epileptogenesis. Activating mutations of the pathway themselves are known to cause a spectrum of neurodevelopmental disorders, including epilepsy. These phenomena highlight common nodes in the form of small-molecule targets, that can be utilized in developing potential therapeutic strategies. With the advent of these new concepts and tools in the epilepsy field, we are presently at an exciting juncture to circumvent the current bottleneck of drug resistance in children and reduce the need for invasive surgeries in order to provide a better quality of life in future patients with pediatric epilepsy.
